# The system wasn’t built for her: an integrative review of women’s experiences in psychiatric and forensic units

**DOI:** 10.3389/fpubh.2026.1810224

**Published:** 2026-06-04

**Authors:** A. Lessard, I. Fettous, C. Cassivi, E. Hudson, Comité Femmes et Psychiatrie du Collectif de défense de droit de la Montérégie, M. Désilets, M. H. Goulet

**Affiliations:** 1 Faculté des Sciences Infirmières, Université de Montréal, Montréal, QC, Canada; 2 Centre de recherche de l’Institut universitaire en santé mentale de Montréal, Montréal, QC, Canada; 3 Faculty of Psychology and Neuroscience, Maastricht University, Maastricht, Netherlands

**Keywords:** forensic, hospitalization, institutional violence, intersectionality, mental health, psychiatry, trauma, women

## Abstract

**Introduction:**

Many studies report that women’s access to safe and equitable care remains limited due to interpersonal and systemic barriers. In mental health care contexts, including psychiatric hospitalization, these inequities are reinforced by the gendered stigmatization of mental health disorders, which influences both diagnosis and clinical interventions.

**Objective:**

The aim of this integrative review is to explore women’s experiences in psychiatric and forensic units. The research questions are: (1) What are the gender-based violence experiences reported by women during psychiatric hospitalization? and (2) What current or potential individual, interpersonal, and systemic initiatives exist to prevent or address gender-based violence?

**Methods:**

An integrative literature review was conducted employing an intersectional approach and lived experience knowledge. Searches across six databases, supplemented by manual searches in the grey literature and reference lists, yielded 54 articles. Thematic analysis was used to analyze data.

**Results:**

The identified themes are: (1) when men are the norm: the making of women’s subordination, (2) the cycle of harm: trauma, systems, and retraumatization, (3) institutional disregard of women’s bodies: reproductive care and maternal role, (4) layered harms: when intersecting identities meet institutional violence and (5) paths forward: initiatives to counter institutional gender violence. The results indicate the existence of hospitalization experiences specific to women that include gender-specific violence and coercion, with experiences in forensic psychiatry care displaying additional specificities related to the legal context and length of the hospitalization.

**Conclusion:**

The results suggest that gendered power dynamics are transferred to the context of psychiatric and forensic hospitalization. Current clinical practices and training do not consider the specific needs of women in these settings. This review underscores the importance of recognizing and addressing the specific needs of women during psychiatric hospitalizations.

## 1 Introduction

Women have always experienced psychiatry in different ways than men. During the nineteenth century, in Western societies the dominant ideal of the “good woman” centered on Whiteness, domesticity, purity, piety, and motherhood, and women who failed to embody these norms were frequently labelled as mentally ill (1, 2). Within this framework, white women were deemed more emotional, impulsive and thus more prone to madness than men, while biological motherhood was paradoxically framed as both the essence of femininity and a potential cause of insanity (1, 2). For Black, Indigenous, and women of color, these gendered constructions intersected with racialist logics that positioned them outside the ideal of womanhood altogether, rendering them either invisible within psychiatric discourse or subject to heightened surveillance, pathologization, and coercion (3, 4).

Psychiatry focused on gendered, bodily explanations, with concepts such as hysteria linking women’s mental disorder to mysterious uterine and ovarian functions. The overrepresentation of women in asylums in the eighteenth and nineteenth centuries has been described as evidence of how these institutions were used to regulate and subjugate women (1, 2). By the late nineteenth century, autobiographical writings by women who experienced involuntary confinement in asylums during this period exposed abuses and contributed to the gradual development of alternatives such as voluntary admission (1). During the 1960s and 1970s, feminist activism intersected with the antipsychiatry movement to challenge psychiatry’s coercive practices and its role in social control, arguing that psychiatric diagnoses policed gender norms and disciplined women who deviated from patriarchal expectations (5, 6). Scholars such as Phyllis Chesler (7) argued that psychiatric diagnosis and institutions served to discipline women and enforce passivity, pathologizing any deviation from patriarchal standards of submissive, dependent womanhood as neurosis or madness. In forensic psychiatry, women were perceived as “double deviant,” in the sense that they were “mad and bad”, pathologized when they violated familial and gender norms, and medically managed into a patriarchal approach to treatment (8).

Nowadays, women’s experiences with mental health services remain shaped by the vestiges of the past. Within mental health services, women are, as service users, more likely to be or to have been victims of different forms of abuse and violence across their lifetime (9). Systematic reviews by Khalifeh et al. (10) and Oram et al. (11) revealed that between 15 and 22% of women living with severe mental disorders have experienced recent sexual violence, and between 30 and 60% of women hospitalized in psychiatry have experienced intimate partner, family or sexual violence over the course of their lives. Gendered stigmatization of mental health disorders influences diagnosis and clinical interventions (12, 13). For example, women are more likely than men to receive prescriptions for antidepressants, anxiolytics or other psychotropic medications, a trend that has only increased in recent years showing that gendered interpretations of symptoms have direct consequences on treatment (14, 15).

For these women, mental health services often function as spaces of disempowerment and re-traumatization due to coercive practices, a lack of trauma-informed approaches and the failure to consider women’s specific needs (16, 17). Women can also be less satisfied by their care as inpatients than men (18). Regarding coercive practices, women are often more critical of these practices than men (19, 104). Their experience of restrictions in inpatient settings can be seen as a reflection of the broader lack of power that women face in society and may mirror abusive life experiences they have previously encountered (20, 105). These negative experiences can in turn lead to mistrust of health systems, reluctance to seek help, and lower engagement in care (17). Instead of promoting recovery, some inpatient psychiatric practices may even reproduce dynamics of trauma and control and thereby worsen gender-based inequities in access to care (16, 21).

In secure forensic settings, clinical information about women’s needs and appropriate treatment strategies also remains limited (22) as women are a minority (23). Criminological theories, as well as forensic assessment tools and treatment programs, were developed and validated mainly on male patterns of offending and risk (23, 24); more attention has been placed on understanding violent behaviours in men than in women (23). Current interventions prioritize risk assessment and management over gender-specific and trauma-informed approaches, despite women in forensic psychiatry presenting high rates of interpersonal vulnerabilities and trauma (22, 23). Thus, women in forensic psychiatry generally receive treatment based on assessments made around male characteristics and needs, undermining their specific life trajectories and pathways into mental illness and offending. Therefore, recent studies provide evidence that women receiving care in forensic psychiatry remain with unmet needs (25).

Taken together, these findings highlight major differences in how men and women experience mental healthcare, as well as the broader inequities in opportunities that shape women’s health trajectories and access to services (26, 27). An increasing number of articles address the experiences of women hospitalized in mental health and forensic settings. Developing a deeper understanding of women’s experiences in these contexts is therefore essential to building care practices that are respectful, equitable and genuinely responsive to their specific needs.

To our knowledge, three existing knowledge syntheses address this topic. In 2009, Määttä (28) published a critical literature review comparing psychiatric hospitalization experiences between women and men. Archer, Lau and Sethi (29) then conducted a review examining the characteristics and needs of women in acute and secure psychiatric units, focusing on identifying gender-specific requirements within these settings. More recently, Scholes et al.’s (30) qualitative systematic review examined the psychiatric hospitalization experiences of women and healthcare providers. However, these knowledge syntheses did not include intervention-focused articles, thereby limiting their ability to inform the development, implementation, and evaluation of practices aimed at transforming psychiatric care for women. Women hospitalized in psychiatric settings and healthcare providers’ perspectives were combined in those reviews, which can overlook underlying power dynamics and inequalities between these diverse sources of information. Despite these knowledge syntheses, women’s gender-specific experiences both in psychiatric and forensic units, particularly regarding gender-based violence and targeted interventions, remain largely underexplored. This review focuses on women’s lived experiences in psychiatric hospitalization, particularly since psychiatric and forensic units often overlap or remain undivided in certain regions.

The aim of this integrative review is to explore women’s experiences in psychiatric and forensic hospitalization contexts. The research questions are: (1) What experiences of gender-based violence are reported by women during psychiatric and forensic hospitalization? and (2) What current or potential individual, interpersonal, and systemic initiatives exist to prevent or address gender-based violence in these settings?

### 1.1 Theoretical framework

This integrative review is grounded on key literature associated with the Intersectional approach (31–37). Intersectionality examines how systems and structures of oppression interact, especially regarding race, gender, social class, disability and other social identities. Treating discrimination as an isolated phenomenon obscures the experiences of women positioned at the convergence of multiple oppressive structures. Intersectionality shows that these forms of oppression do not merely accumulate additively but rather intersect and manifest in distinctive ways, creating a unique standpoint.

## 2 Methodology

### 2.1 Positionality statement

We consider that identifying yourself as a woman and having a mental illness that required inpatient care is an identity intersection, a position held by several of the review’s authors. The first author (AL), EH, and the Women and Psychiatry Committee of the Montérégie Rights Defense Collective (C*omité Femmes et Psychiatrie du Collectif de défense des droits de la Montérégie*) are all women with lived experience of mental health issues and psychiatric hospitalizations; the latter two (EH and Committee) were invited to contribute at different stages of the review and to confirm consistency of the results with their own experience. According to Borkman (1976) (35–38), experiential knowledge, derived from lived experiences and one’s intimate understanding of their own reality and situation, constitutes a unique form of expertise. This literature synthesis acknowledges the contribution of experiential knowledge alongside more traditional sources of knowledge such as theoretical and clinical knowledge.

### 2.2 Methodological approach

The approach by Whittemore and Knafl (39) and the work by Toronto and Remington (40) served as references for conducting this integrative review. The integrative review was chosen as a knowledge synthesis approach because it allows the inclusion of diverse research methodologies to obtain a more complete and rigorous understanding of women’s experiences in psychiatric hospitalization contexts (39). The integrative review can also be conducive to integrating experiential knowledge throughout its various stages, from problem identification to results interpretation (41). The main stages of an integrative review include: (1) problem identification, (2) literature search, (3) quality appraisal of studies, (4) data analysis, and (5) presentation of results.

### 2.3 Problem identification

The research question for this article was identified through collaboration between our research team and the Women and Psychiatry Committee of the Montérégie Rights Defense Collective. The Montérégie Rights Defense Collective (CDDM) is a regional community organization that advocates for the rights of people who are experiencing or have experienced mental health issues. In 2024, the Committee brought together women with experience of psychiatric hospitalization in one specific administrative region of Quebec (Canada) to answer the following question: “Where does women’s power lie?” This discussion group led to the creation of a partnership with our research team to better understand women’s experiences in psychiatry. Following a review of existing articles on gender-based violence, we jointly identified a gap in the literature, leading to the development of this review.

In this article, “woman” encompasses all individuals who identify or have previously identified as woman as well as individuals whose experiences were shaped by feminine attributes. This definition was developed by multiple discussions and reflections with the Women and Psychiatry Committee. This co-constructed definition aims to be inclusive but likely does not cover the breadth of every experience. To analyze gender-based violence, we had to define the concept. The European Institute of Gender Inequality (42) defines gendered-based violence as “a phenomenon deeply rooted in gender inequality and continues to be one of the most notable human rights violations within all societies”. More broadly, violence is understood as the World Health Organization’s (WHO) (43) as “the intentional use of physical force or power, threatened or actual, against oneself, another person, or against a group or community, that either results in or has a high likelihood of resulting in injury, death, psychological harm, maldevelopment, or deprivation.”

## 3 Literature search

The literature search was conducted in April 2025. The development of the conceptual framework and search strategies was carried out in close collaboration with a librarian specializing in mental health. The key concepts targeted in the search were: women and women-related issues, and psychiatric hospitalization’s experiences. The search strategies were subsequently peer-reviewed and validated by a second librarian using the Peer Review of Electronic Search Strategies checklist (see Supplementary material for the search strategy). The search was executed across six databases: PubMed, Medline, Embase, PsycINFO, the Cochrane Database, and CINAHL. The types of documents included in the review comprised journal articles, theses, books, and book chapters. Additionally, grey literature was sourced from academic and institutional websites, references of 30 previously identified articles, as well as ResearchGate. Grey literature encompasses all documents that are not controlled by commercial publishers.

**Figure 1:**
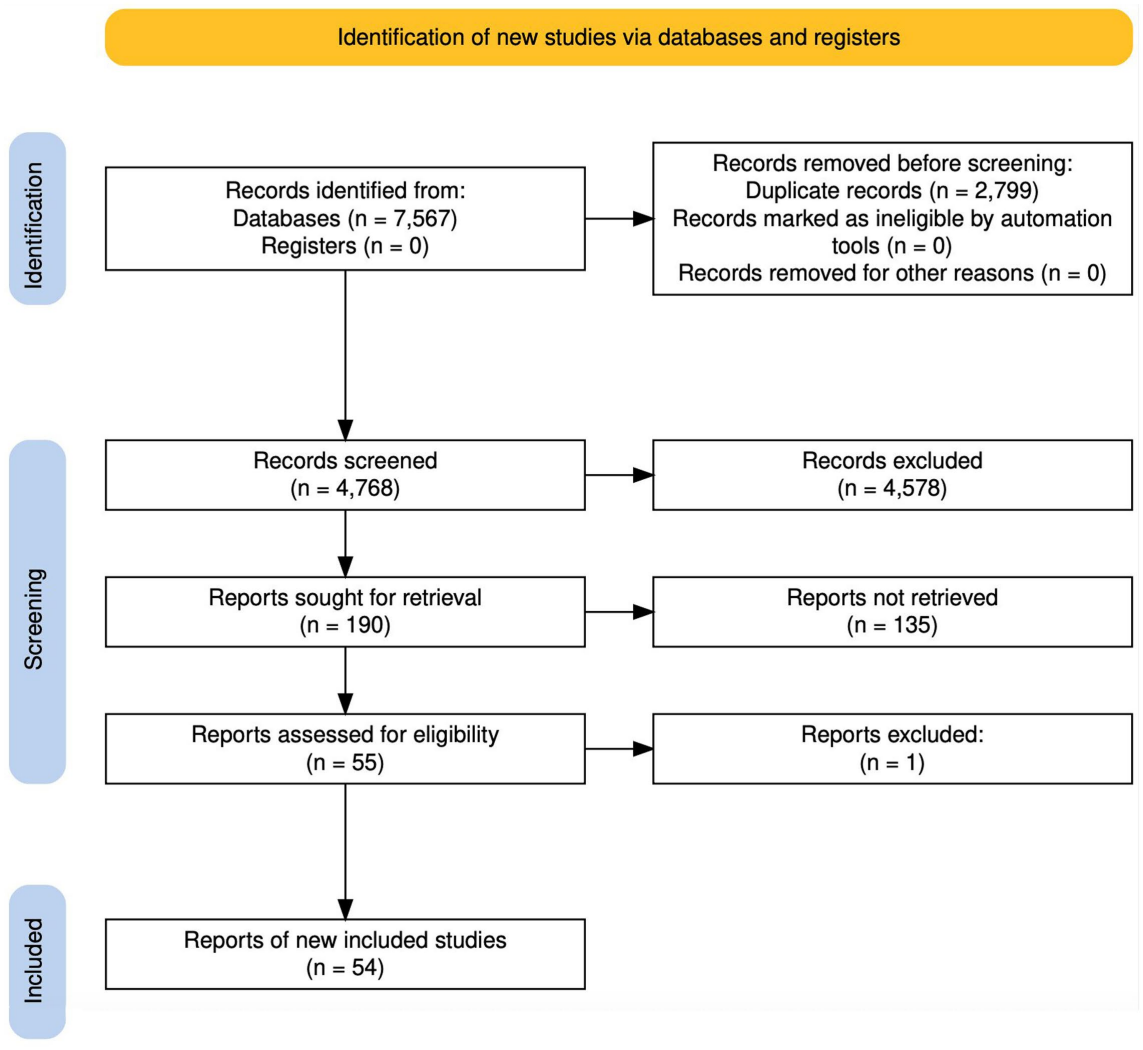
Flow diagram describing the search strategy employed for the inclusion of the 54 articles featured in the integrative review.

7,567 records were identified. Following the database searches and de-duplication using SR Accelerator (which removed 2,799 duplicates), a total of 4,767 articles were imported into Covidence for screening and data management. The selection process began with an inter-rater agreement of 100% for a random sample of 1% of the articles (*n* = 48), screened by three other reviewers (IF, CC, and EH). 189 articles were selected for full text review. An inter-rater agreement was done again for a random sample of 10% of the articles (*n* = 19). The inter-rater agreement reached 80% between the three reviewers. Any discrepancies in selection were discussed between the two reviewers until a consensus was reached. If no consensus was obtained, a third reviewer was involved to make a final decision. At the end, 54 articles were included in the data extraction of this integrative review. The full screening process is resumed in a PRISMA flow diagram (Figure 1).

Psychiatric and forensic units were purposefully selected for this review due to their shared legal frameworks governing patient admission and treatment, including voluntary status, treatment orders, and involuntary hospitalization protocols. These contexts distinguish them from carceral environments that were excluded due to their punitive orientation rather than therapeutic intent. Specialized settings blending physical and mental health care, such as eating disorder units, were likewise omitted to maintain emphasis on environments where psychiatric intervention predominates. The following inclusion and exclusion criteria were used to guide the selection. No inclusion or exclusion criteria based on the geographical location of the included studies were adopted, with the aim of not excluding any experiences that could enrich an intersectional analysis. These criteria were applied rigorously during the screening process to ensure alignment with the objectives of this integrative review (Table 1).

**Table 1:** Table summarizing the criteria applied during the screening process.

Type of criterion	Explanation of criteria applied
Inclusion criteria	Literature where the experiences of women was the central focus, either through women-only samples or through mixed-participant samples (men or staff, provided that women’s experiences were clearly identifiable and reported separately) Literature examining psychiatric hospitalization in adult psychiatric or forensic settings. Qualitative studies, quantitative studies, mixed-method studies and theoretical or conceptual papers Literature published in French or English Literature published between 2000 and 2025
Exclusion criteria	Literature focused solely on the perspectives of healthcare workers (e.g., staff, nurses) or exclusively on men rather than on women’s patient experiences Literature in other healthcare settings (perinatal/mother–infant units, physical healthcare, community, outpatient, substance use, eating disorders, oncology, perinatal care, veteran/military, intellectual disability, correctional facilities, telepsychiatry) or focusing on populations outside general adult psychiatry (children, adolescents, older adults) Literature published in languages other than French and English Literature published before 2000

### 3.1 Quality appraisal

As recommended by Whittemore and Knafl (39) and Toronto and Remington (40), a dichotomous scoring system (1 = low quality, 2 = high quality) was used by AL and IF to assess each study. However, no studies were excluded based on their quality scores; instead, quality assessments were used to contextualize the integrative synthesis of the data and interpret heterogeneity of the findings.

### 3.2 Data analysis

A data extraction form was created in Covidence that included the following elements: author names, article title, year of publication, country of origin, study design, data collection methods, research aim, study population, sample size, summary of key findings, study limitations, quality appraisal score, as well as a section capturing reflective insights and salient elements noted during extraction. This final section was integrated to center experiential knowledge of those involved in the review process, ensuring that the synthesis remained coherent with the lived experiences of women hospitalized in psychiatric settings.

Data analysis followed the six steps of Braun and Clark’s (44) thematic analysis approach: immersing oneself in the data, producing initial codes, aggregating themes, defining themes, and writing the report. An inductive coding approach was used for this thematic analysis, facilitated by qualitative data analysis software. Quantitative data were transformed into descriptive categories for thematic analysis. Initial codes were produced with Libre QDA software (version 1.0). After theme aggregation, a thematic analysis workshop was conducted with members of the Women and Psychiatry Committee. This activity was designed using tools from collaborative thematic analysis, the round robin method (45), and the *Participatory Research Toolkit for Social Norms Measurement* from United Nations Children’s Fund (46). During this workshop, the women were able to share their knowledge, validate the importance of certain themes, and redefine others. This exchange reinforced the thematic analysis and shaped the following results, with the hope that they more fully reflect the realities of women hospitalized in psychiatry.

## 4 Results

A total of 54 articles were selected for this review: 9 knowledge syntheses, 2 government publications, 7 quantitative studies, 6 mixed method studies, 22 qualitative studies and 8 other types (lived experience narrative opinion/editorial, commentary articles, theorical article, book or book’s chapter, thesis). 52 of the 54 articles are in English and 2 are in French. The majority of the articles are from the United Kingdom (*n* = 19, mostly forensic psychiatry), Australia (*n* = 13), Canada (*n* = 5) and the United States (*n* = 4). The other countries are Sweden (*n* = 2), Ireland (*n* = 2), Norway (*n* = 2), Germany (*n* = 1), United Arab Emirates (*n* = 1), New Zealand (*n* = 1), France (*n* = 1), Greece (*n* = 1), Czech Republic (*n* = 1) and Brazil (*n* = 1). Of the 54 articles, 47 were written by someone who identified as a woman as first author, 6 written by someone who identified as a man as first author and 1 written by someone who identified as non-binary or gender diverse.

The studies identified five themes included: (1) “when men are the norm: the making of women’s subordination”, (2) “the cycle of harm: trauma, systems, and retraumatization”, (3) “institutional disregard of women’s bodies: reproductive care and maternal role”, (4) “layered harms: when intersecting identities meet institutional violence”, and (5) “paths forward: initiatives to counter institutional gender violence” (see Table 2 for a list of themes and subthemes).

**Table 2:** Themes.

Themes	Subthemes
1: When men are the norm: the making of women’s subordination	1.1: The advantages of being a man 1.2: The infantilization of women 1.3: The loss of credibility 1.4: The expectations of gender
2: The cycle of harm: trauma, systems and retraumatization	2.1 Women’s trauma and retraumatization 2.2: System responses to women’s trauma
3: Institutional disregard of women’s bodies: reproductive care and maternal role	3.1: Menstruation 3.2: Hormonal health overlooked and mislabeled 3.3: Parental role and perinatal care dismissed
4: Layered harms: when intersecting identities meet institutional violence	4.1: Cumulative stress and microaggressions experienced by SLGBTQIA+ women 4.2: Disparities among racialized women 4.3: Indigenous women and institutional harm
5: Paths forward: initiatives to counter institutional gender violence	5.1: Submission and revolt 5.2: Women’s solidarity and dissent 5.3: Physical setting and staff composition 5.4: Therapeutic activities 5.5: Integrating women’s voices 5.6: Trauma-informed and intersectional training

### 4.1 Theme one: when men are the norm: the making of women’s subordination

#### 4.1.1 The advantages of being a man

From a structural perspective, Cutting and Henderson (47) argue that women’s psychiatric care experiences are embedded within patriarchal narratives and environments where masculine ways of being are privileged and can even be considered the norm. Women’s narratives consistently highlight that behavioural practices on psychiatric units appear to privilege male conduct, rendering it more socially acceptable than equivalent female behaviours (48, 49). When men act out, their behaviours appear to be taken more seriously by staff, prompting swift and authoritative responses, in contrast to women’s expressions which are often minimized (29, 49, 50, 101).

*“It feels that ward staff are so used to treating women and girls badly that they are shocked when this type abuse gets called out for what it is, and it us clear abuse”* [(51), p.6].

Literature reveals a perception of differential treatment on psychiatric units, where male patients appear to have greater privileges compared to their female counterparts (28, 49). Specifically, women report feeling disadvantaged relative to men, who secure expedited access to privileges such as going outside the unit more quickly, despite comparable clinical conditions (49, 52).

One woman said “*I do think that my attitude or attitude problem, as some call it, would be tolerated a lot more if I was a man. I do think the word “bossy” comes to mind [to] some people [when they] describe me, but I do think men get away with it, being called more “assertive” than “bossy” and I think with men it’s just expected more, this kind of hot head if you like. You know I generally feel that […] men get away with a lot more of that kind of behaviour than women”* [(53), p.33].

#### 4.1.2 The infantilization of women

Hospitalized women in psychiatric units report experiences in which they felt infantilized by staff (49, 52, 54). Women describe feelings of diminishment, constantly required to justify their every action and treated like children (48, 52). Staff interactions, including tone, vocabulary, actions, and nonverbal behaviours, shape women’s perceptions of respect and rights recognition during psychiatric hospitalization (48, 49). In the Action Autonomie report (48), a woman said, “*I felt infantilized by the tone the healthcare staff used towards me […], I felt like a second-class citizen*”. They experience powerlessness over themselves and their care and violations of consent (48, 51).

#### 4.1.3 The loss of credibility

Women hospitalized on psychiatric units frequently encounter a profound loss of credibility, where their voices, testimonies, and self-reports are systematically invalidated or dismissed by clinical staff (52).

*“The staff are not treating me with ‘sincerity’, and they roll their eyes back, which is a definite body language … I just, I don’t feel like I wanna participate … when things like that happen”* [(52), p.299].

This manifests through assumptions that their accounts, particularly of trauma, mistreatment, or rights violations are delusions, exaggerations, or manipulative behaviours stemming from their diagnosed mental illness. This undermines autonomy, discourages disclosure of legitimate concerns and perpetuates power imbalances (48).

*“You don’t need to be mentally ill; you are a woman, nothing. As a woman, your credibility is much, much lower. And as a woman, you’ll have to perform five times harder to earn respect in an environment where there are often more men—it feels like no one believes you”* [(48), p.38].

#### 4.1.4 The expectations of gender

Women face gendered expectations that directly influence diagnostic practices, where deviations from normative femininity are pathologized as psychiatric symptoms (48, 49, 52). Participants report that assertive self-expression or perceived hypersexuality, such as wearing a short skirt or “too much makeup”, triggers labels of mania:

*“A lot of my admissions, like the ‘manic’ related ones were related to their perception of me as being overly confident and also hypersexual … they made all these comments about my skirt being too short”* [(54), p.653].

Similarly, women engage in constant self-monitoring to avoid personality disorder diagnoses when asserting boundaries (29, 48).

*“I was experiencing massive overwhelming grief. And that’s what I needed support with. But it was very much pathologised … I got labelled with borderline personality disorder, and it was pretty much all downhill from there…. I have very big issues with the term manipulative. Like I’ve been told I’m manipulative about a thousand trillion times, and yes I refer to it as desperate help seeking behaviour”* [(54), p.654].

Staff expectations of “appropriate” feminine presentation further enforce compliance (49, 50). These dynamics reveal how gendered norms shape diagnosis, transforming women’s agency into clinical pathology (49, 54). In forensic psychiatry, patriarchal norms seem to impact women’s sense of identity.

A woman said *“I don’t think you’re seen for the person you are either, and are treated in that way, instead you are supposed to be like a robot in the way they expect you to be, it’s not possible for the whole person to be who one is, humble, sensitive, kind, you have to be tough but at the same time being honorable towards them, the sensitivity that we woman have is suppressed in one kind of way, that’s how I feel”* [(55), p. 819].

Hospitalized women are not expected to take up the same space as men, presenting projected “womanly” traits such as being soft and calm (55). However, women are also expected to adapt to “manly” norms on the ward such as being tough and communicating directly. Thus, women report that the normative expectation is that they should adapt to a male-centered environment, without letting go of their projected female characteristics.

### 4.2 Theme 2: the cycle of harm: trauma, systems, and retraumatization

#### 4.2.1 Women’s trauma and retraumatization

The literature reveals the prevalence of gender-based violence in the life histories of women hospitalized in psychiatric units (56).

*“(She) felt that she had been treated badly by life. She blamed this on events during her childhood: sexual abuse by men, including an incestuous relationship with her father, abandonment, and a lack of understanding. All of these events have left her feeling powerless and without choices. For her, life’s circumstances—identified as an overload of emotional torment, which were reflected in her behaviour—have influenced her hospitalization. After almost 50 years of living in a hospital, she says”…but I am not crazy. I am mentally ill”* [(57), p.128].

Multiple studies highlight women’s fears regarding their safety on psychiatric units. Women express apprehension during hospitalization about various forms of violence, including physical, sexual, and verbal assaults (Department of Health & Social Care) (28, 54, 56, 58).

Women’s fears for their safety intensify during sleep, when they feel particularly vulnerable and less able to defend themselves (25). Both patients and staff can evoke these fears in women hospitalized in psychiatric units, particularly male patients or male staff for some women (54, 59, 60).

*“She had been abused and she found it difficult on the ward, she was frightened of men and there being men around”* [(47), p. 710].

*“As a person that has experienced sexual abuse…. A petite, 19-year old girl, being held down by three large, male, security guards whilst my clothes were removed so they could inject a sedative into my rump…. I was of the belief that I was being taken into a room to be raped. The situation had scarily escalated, I cannot really recall how it ended up being so violent … the trauma from that incident is something I have had to work with ever since”* [(54), p.653].

Furthermore, in forensics settings, women are often faced with the use of seclusion and restraint which can result in the exacerbation of existing trauma responses (30). Despite seclusion and restraint being used in many psychiatric settings, this aspect has been more extensively discussed in forensic settings where women describe these experiences as negative and traumatizing. Studies have demonstrated that these restrictive practices often result in the exacerbation of existing trauma from past experiences in women’s lives:

*“When they restrain you they hold you down and all that’s going through my head is angry and abused again … it brings back bad memories for me”* [(61), p.13].

#### 4.2.2 System responses to women’s trauma

Women hospitalized on psychiatric units frequently face institutional responses that ignore, or trivialize their reported experiences of distress, trauma, or mistreatment, or blame (43, 62). Patients have described that staff may dismiss legitimate complaints as psychiatric symptoms, shift responsibility onto patients through victim-blaming narratives or normalize abusive dynamics as routine (54, 59).

*“I knew how they were labeling me or how they said they were trying to treat me, but I didn’t feel what they were saying fit with what I had experienced”* [(56), p.53].

However, it remains largely unaddressed in clinical practice: childhood abuse, familial violence and intimate partner violence can be disregarded as irrelevant to current mental health presentation or inappropriately medicalized, framed as symptoms of individual psychopathology rather than as traumatic exposures requiring trauma-informed care (25, 54, 58).

*“And that’s what I got angry for and frustrated for at the end, because it took me 48 years to be heard and yet I’d been shouting from about [the age of 18 years old] and never got heard… I’ve been telling [people] it about a hundred times… but nobody’s ever actually picked it up or my behaviours [as] a result of things. Nobody ever bothered to ask me ‘why are you doing these things?” or ‘why is this happening to you?’, but if they had asked me they would have found it was all down to sexual trauma, everything was down to sexual trauma”* (59).

This systemic neglect contributes to misdiagnosis, inadequate treatment planning, and perpetuation of revictimization within institutional settings. This can manifest through coercive practices, interpersonal invalidation by staff, environmental triggers reminiscent of abusive dynamics, or witnessing violence against other patients (49, 56).

##### 4.2.2.1 Coercive practices

Women can experience coercive practices differently than men (63–65). Experiences of coercive practices were mentioned by women as being scary and traumatizing, even more when male staff was involved (66, 67). In forensic psychiatric settings, most women express witnessing physical assault or threats and report exposure to staff assault, restraint, interpatient violence, verbal abuse and sexual exhibitionism, perpetrated by male patients (68).

*“I have been hit by another patient, the same one who wanted to have sex with me in the showers. When the male patients kick off, then I do tend to be scared of them. They’re much more violent and aggressive”* [(68), p.580].

Moreover, women, from psychiatric and forensic units, perceive restrictive practices as a form of punishment administered by the caregivers (6, 30, 61). Often, patients feel that the degree of seriousness of the behaviour does not match the intensity of the negative experience during restraint:

*“We should have been treated equally. I don’t think it’s right you let one patient off and then punish another. All I see is they punished me for just running up and down stairs”* [(61), p.13].

On rarer occasions, particularly in forensic settings, restraint has been documented as being experienced as a form of containment, evoking feelings of comfort and safety. Some women describe that it created a predictable outcome:

*“Every time they let go I started again. It was over half an hour struggling…Made me feel like safe and comfortable. Make sure that nobody hurt me apart from them making me feel safe”* [(61), p.14].

However, these feelings arose mainly when female staff members applied the restraint, and the feelings of containment were ultimately paired with anger and anxiety.

Furthermore, Nawka et al. (63) mentions that chemical restraint may be used more frequently for women with schizophrenia in psychiatric settings. Some reports describe women’s fear of chemical restraints, as they felt “drugged” with or without consent and worried about potential consequences while their faculties were impaired (69).

*“I was quickly admitted to the psychiatric ED and placed in a room with seven men, positioned beneath a window that was partially obscured from the nurse’s station. I was stripped of my clothes and personal belongings and was heavily sedated. When handed a cocktail of pills, I didn’t ask questions; I just swallowed them and fell asleep. At times, I voiced concerns about being assaulted in the room, something I had witnessed happen to patients in my clinical practice. I feared that in my semi-comatose state I was particularly vulnerable; however, this worry was repeatedly dismissed, and for 2days, I lay in bed, semi-conscious, saying nothing, doing nothing”* [(69), p.870].

### 4.3 Theme 3: institutional disregard of women’s bodies: reproductive care and maternal role

#### 4.3.1 Menstruation

The relationship between physical and mental health seems to remain insufficiently acknowledged within psychiatric care for women, particularly regarding women’s reproductive health. Issues related to contraception are frequently neglected (70).

*“They messed up my pill regularly, ran out of it, forgot to order, causing my PMDD to hugely worsen which lead to suicide attempts”* [(51), p. 5].

The experience of menstruation among women hospitalized in psychiatric units is frequently marked by restricted access to adequate menstrual hygiene products and disposal; products are often limited in options, locked away, rationed, or available only upon request to staff, forcing patients to disclose intimate needs repeatedly and sometimes to rely on inadequate alternatives such as tissue or prolonged use of the same pad (51, 52). In Porter (51), women revealed that staff in low secure settings would stop patients from using period products due to risk factors, but this left many patients in dehumanising embarrassment. The lack of access to menstrual products in psychiatric settings is closely linked to feelings of humiliation, loss of dignity, and a broader lack of consideration for women’s fundamental needs (51, 52).

*“When I was in HDU I had no Pink Lady or pad sanitary bin and basically I had to keep going up to them with it in a toilet wrapper*” [(52), p. 299].

Such practices undermine patients’ dignity, erode their sense of bodily autonomy, and can be understood as a form of institutional violence that signals that their menstrual health is secondary to safety or organizational convenience (51). Pharmacological and non-pharmacological options to manage menstrual pain can also be difficult to access (51). The psychological harm of disregarded menstruation transcends physical discomfort, epitomizing the pervasive invisibility of women’s reproductive health within psychiatric care (51, 60, 71).

#### 4.3.2 Hormonal health overlooked and mislabeled

Hormonal health receives inadequate attention in psychiatric inpatient settings, particularly for transgender individuals and cisgender women experiencing cyclical symptoms (51, 70, 72). Transgender people can face denial of hormone therapy during hospitalization, with clinicians attributing mania or instability to gender-affirming treatment rather than contextualizing it within transition care:

*“They [inpatient psychiatrist] wouldn’t give me T [testosterone], cause they decided that… Maybe that’s why I was manic…He’s actually still my psychiatrist. He’s really nice. But he fucked up. He’s apologized but…it’s 2000 fucking17…and…they are withholding my hormones…”* [(70), p. 6].

Similarly, hormonal cycles are frequently dismissed or mislabeled as standalone psychiatric symptoms, despite patient and family reports of mental health crises coinciding with menstrual phases and subsequent improvement (71). Noteworthy, no article was found about menopause and perimenopause, a hormonal state that every person with a uterus goes through. This pattern reveals a tendency to pathologize or ignore endocrine fluctuations from gender transition or menstrual cycles, rather than integrating hormonal care into holistic psychiatric treatment (71).

#### 4.3.3 Parental role and perinatal care dismissed

In forensic psychiatric settings, hospitalizations are often longer than general psychiatric settings, ranging from months to years. Consequently, it is not uncommon for women to feel a desire to start a family during their hospitalization. However, women report feeling frustrated and worried this might not happen due to prolonged hospitalization during their “reproductive” years:

*“Well I’m 34, it is time for me too, it’s not only that I feel a longing, I also feel the importance of the biological clock and that makes me feel stuck like I don’t even know if I will get a baby. And that the forensic psychiatry has taken my life away from me, it feels that way that it could possibly be like that. And that’s like taking away the finest thing in a human life”* [(55), p.819].

Evidence on the treatment of women facing mental health challenges during the perinatal period remains limited (49, 60, 71). Beyond this issue, doubting women’s parental capacities and discouraging motherhood remains common (48, 54, 73). For women with parental responsibilities, the fear of losing children can be a significant barrier to seeking help. Child removal can surpass other traumas like substance use, incarceration or hospitalization in severity (73). Motherhood is often considered a vital source of purpose; however, pregnancies may be viewed by clinical teams as a source of inevitable irresponsibility, violence, or clinical deterioration (73): “*Nobody seemed happy when I said that I was pregnant*.” She said that other people considered that she would not be able to raise her child, that she would be a “*violent*” or “*irresponsible*” mother and that motherhood would aggravate her mental problems [(73), p.396]. Parental roles are routinely disregarded or leveraged punitively, including bans on child contact or bonding during inpatient stays despite breastfeeding commitments and beneficial effects for women (54, 71). Women may strategically evade admission to safeguard custody, while staff deploy threats of permanent separation or lifelong medication to erode self-efficacy (49, 54, 56). Such practices frame mental illness as antithetical to parenting, prioritizing institutional protocols over support for dual maternal and recovery roles (49).

*“Yeah from my daughter d’y’know what I mean from bonding with her and I just feel like it’s tight like I feel like she needs her Mum right now…I’m her birth mum d’y’know what I mean so she needs to bond with me”* [(49), p. 116–117].

In forensic psychiatry, parent patients at admission present different life course trajectories than patients without children. Parent patients are often less likely to have a diagnosis of comorbidity than non-parent patients; however, their index offense is more likely to be violent and within their social circle. Most parents lose the custody of their child upon admission in a forensic setting. Furthermore, minors often lose contact with their parents upon admission in a forensic ward, while adult children tend to maintain contact with their parents (74). However, in general psychiatric and forensic settings, women often describe their interpersonal relationships as meaningful and crucial to their recovery. Women report that a social circle after discharge allows them to reintegrate society successfully (56).

*“And he [the psychiatrist] said, ‘You will never reintegrate into society,’ and told me I’d probably never hold a job and that it would be unlikely for me to get custody of my daughter again. He threatened that I wouldn’t have my daughter back and that I would never be able to go to a normal school. And that I would be on medication for the rest of my life. This is what he told me. I was crushed. I could not believe it”* [(56), p.53].

### 4.4 Theme 4: layered harms: when intersecting identities meet institutional violence

*“I’ve always been involuntary admitted to a psychiatric hospital and always in the back of a paddy wagon in handcuffs …all those experiences have been so traumatising and that. And just sitting there and having so many police surround you … it’s horrific, that level of not treating you as a human being, because I’m not a white girl”* [(54), p.654].

Of the 54 articles, only 11 articles included sociodemographic data about the racial identity, Indigenous status, gender, or sexual orientation of the sample. Mistrust toward the psychiatric system and its staff also appears to intensify according to their experiences of discrimination, and the burden of these experiences seems to be felt acutely by these women (70, 75). Women with intersectional identities are more strongly impacted by the structural power of the psychiatric system (76). The literature also pointed to a gap in service provider knowledge about the specific needs and experience of women at the intersection of marginalized identities (70, 74, 75). These experiences are cumulatively harmful and have significant repercussions for the women hospitalized in a psychiatric unit (77). Furthermore, when women hospitalized in psychiatric settings occupy multiple intersecting identities, the need to maintain connections with loved ones and their communities becomes even more critical (25, 70, 78). An Indigenous woman said that “*Connection-bond with kin, culture and community”* was one the main factor of recovery for her (78).

#### 4.4.1 Cumulative stress and microaggressions experienced by SLGBTQIA+ people

Two-Spirit, Lesbian, Gay, Bisexual, Transgender, Queer or Questioning, Intersex, and Asexual, with the plus sign representing additional sexual orientations and gender identities (SLGBTQIA +) people are susceptible to live different forms of microaggressions during psychiatric hospitalization. Microaggressions can manifest as micro assaults, microinsults and/or microinvalidations (70, 76). Micro assaults are deliberate harmful acts such as name-calling or avoidance. Microinsults involve comments or behaviours that convey insensitivity or covert disrespect. Microinvalidations are communications that dismiss or undermine an individual’s thoughts, feelings, or lived experiences (71, 76).

*“It was really hard… calling around and realizing that you had to ask that question, that if I was hetero[sexual], I would have never had to bring this up…”* [(76), p.1137].

The pathologization of gender and sexual orientation was addressed (70). SLGBTQIA+ people are subjected to minority stress during psychiatric hospitalizations, and this impacts the care that they receive. Minority stress was originally formulated to explain increased mental distress and mental health disorders, including suicidality and self-harm, among lesbian, gay, and bisexual individuals, and this theory has since been extended to gender minorities. It highlights how distal stressors such as gender-related victimization and non-affirmation, combined with proximal factors like internalized stigmatization and anticipation of future discrimination, contribute to adverse mental and physical health outcomes (79). Furthermore, SLGBTQIA+ community members are subjected to gender essentialism as structural violence in psychiatric systems which can lead to stigmatization and to have adverse effects on their psychological health (70, 71).

#### 4.4.2 Disparities among racialized women

The authors of this article acknowledge that the term “racialized women” has been questioned in the literature, but it remains widely used. In this article, “racialized women” encompass anyone who identifies as a woman (consistent with our definition of the concept of woman above) and who experiences systemic racism. Racialized women can experience the strong impact of power imbalances in psychiatric settings and be sensitive to authoritarian relationships and structures (62). Creswell (25) mentions that Black American women hospitalized in psychiatric units felt isolated from their family and their community and it could impact their recovery. They are confronted with more barriers to help seeking and recovery such as housing issues, lack of sensitivity, lack cultural safety and knowledge from care providers and others different forms of discrimination based on their diverse marginalized identities (25, 58, 75).

#### 4.4.3 Indigenous women and institutional harm

There is a lack of cultural safety reported by Indigenous women hospitalized in a psychiatric unit. For example, women reported that staff did not take into account that English was not their first language: they spoke very quickly, without considering whether the women were able to understand the conversation or not (79). The intersection of discriminations lived by Indigenous women seems to extend in the psychiatric system (79): “a Prison of Disempowerment… built out of the lies of colonization” [(80), p. 143 in (79)]. Hospitalization perpetuates the use of prisons and mental health institutions to enable social control and the persistence of colonial structures (81). The fear of being hospitalized in a psychiatric unit can be exacerbated for Indigenous women by these historical patterns of being removed from their family and community for some kind of institutionalization (79). *“White people were boss. Nurses and Doc were God* (78). For example, it can be as simple as language barriers, slowing the speech speed and it can impact the experiences of Indigenous women hospitalized in psychiatric units (78).

#### 4.4.4 Socio-economic status for secure settings

In forensic psychiatry, similar inequities impact women’s experiences in the healthcare setting. Certain factors prior to hospitalization have a significant impact on the experience in secure mental health settings. Research describes violence or abuse as the most prevalent inequalities to affect women (82). Furthermore, socioeconomic inequalities were identified, highlighting school or employment as a protective factor against the development of mental illness. Along those lines, precarious socioeconomic context has been identified as a contributing factor in impacting the experience women will have in secure mental health settings (82). Together with the factors related to general psychiatry, these elements contribute to the overall experience of secure mental health settings.

### 4.5 Theme 5: path forward: initiatives to counter institutional gender violence

#### 4.5.1 Submission and revolt

Women in psychiatric inpatient settings exhibit responses to institutional violence and coercion that oscillate between resigned submission and active revolt (29, 35, 47, 54). Submission often emerges from repeated experiences of dismissal, where patients comply with medication and restrictions because resistance appears pointless, leading to passive endurance of treatment protocols (49, 52). “*I: So what’s changed now, what’s kind of stopped you fighting it now? P: ‘Cause there’s no point…I just go along with it. Just go along, take me meds…*” [(49), p.116]. Participants also highlighted feeling compelled to submit to the environment, treatments, and staff to “survive” their hospitalization, making as few waves as possible, and remaining compliant (35, 83).

Revolt, conversely, can be triggered by coercive practices like seclusion and restraints. Women describe these experiences as radicalizing rather than calming, exposing gender-based power disparities and fueling determination to challenge systemic abuses (54, 76, 79). These polarized reactions underscore how coercive practices may suppress immediate dissent while cultivating deeper resistance, complicating long-term therapeutic engagement (29, 54).

*“Being put in seclusion radicalised me. It didn’t calm me, it radicalised me.… Three or four males trying to overpower a woman. I mean, it’s ridiculous”* [(54), p.653].

#### 4.5.2 Women’s solidarity and dissent

In reaction to institutional harms, certain women embrace solidarity, whereas others disengage from it. Solidarity among women in psychiatric inpatient settings often manifests as robust peer support networks that can surpass formal staff interventions, with patients collectively monitoring each other’s emotional states, providing crisis intervention and offering sustained companionship during distress (25, 47, 56). This mutual aid emerges organically as women “looking out for each other,” creating parallel care systems that prioritize relational empathy over institutional detachment (29, 49). Such bonds counteract isolation, foster resilience and enable collective navigation of hostile environments where official support proves inadequate (29, 47, 49).

*We just look out for each other. If someone’s gonna do something, if we know someone’s down and they are having a bad day, we’d be there for them and we’d sit and talk to them. We do more than what the staff do”* [(49), p.117].

Conversely, disunity arises from individualized survival strategies or internalized hierarchies that fragment group cohesion under institutional pressure (83). Some women may withdraw into self-preservation, compete for limited resources like staff attention or distance themselves from peers perceived as “more ill” or not trustworthy, perpetuating division rather than unity (54, 56). These tensions highlight how psychiatric systems can undermine solidarity, turning potential allies into rivals and weakening the collective power needed to challenge coercive practices (29, 53).

#### 4.5.3 Physical setting and staff composition

Different perspectives exist regarding mixed-sex versus single-sex wards for women in psychiatric settings. The term ‘sex’ is used in this context instead of ‘gender’ because most units seem to use assigned sex at birth as the basis for classification, which creates a structural issue for gender-diverse people. Proponents of mixed-sex wards argue that they promote a sense of “normality” through social interaction, whereas single-sex wards may foster disruption among women or exclusion of transgender patients (53, 84). However, mixed wards heighten perceptions of aggression, particularly among women who fear male intrusion into bedrooms or bathrooms (53, 84). On mixed-sex forensic wards, women commonly report feeling intimidated due to being outnumbered by male patients: *“In the garden. there’ll be like four or five boys sitting there, but it’s the only place to go’* [(68), p.580]. However, when questioned about moving to a forensic single-sex ward, women often explain that their feeling of unsafeness is related to the whole group of patients on the ward, rather than solely men (68).

On forensic single-sex wards, women tend to report less sexual abuse or harassment than on mixed-gender wards. Nevertheless, bullying, intimidation and aggressive behaviours are commonly reported (68). Witnessing these incidents or being directly victimized in this environment fosters an atmosphere of tension, where each individual remains in a continuous state of vigilance, anticipating the next threat. Indeed, most women in the included studies preferred to be in a mixed-sex unit for diverse reasons: from community representativeness to feelings of safety enhanced by having men around.

Women-only spaces in psychiatric units prioritize safety for women, especially those with trauma histories, by mitigating risks of harassment, sexual violence and retraumatization prevalent in mixed-gender environments (27, 29, 83). Another proposed option, though underexplored in practice, involves mixed wards with dedicated women’s areas such as lounges (83, 102). Space management in psychiatric wards must also accommodate transgender and non-binary people to ensure inclusivity (70). Beyond the debate on single-sex versus mixed-sex units, very little information was found in the literature regarding the physical layout of psychiatric and forensic units and women’s perceptions thereof.

For staff gender preferences, assigning only female staff can strengthen trust bonds, as women patients often feel safer disclosing trauma histories, reporting abuse or engaging in care without fear of male authority figures triggering past violations (27, 48, 85). This gender-matching may reduce coercion in psychiatric units, enhances therapeutic alliances and counters historical power imbalances, with studies showing improved outcomes when women perceive staff as empathetic allies rather than institutional enforcers (22, 47, 86). In forensic units, women reported feeling comfortable with the presence of male staff, as some explain that the presence of male staff enhanced their feeling of security.

*“I don’t feel safe in here at all, people bringing razor blades in, people attacking me, people attacking people all the time. Punching them in the head, kicking them, scratching them. I don’t feel safe with the kind of patients that we’ve got on the unit at all. The kind of patients they’re bringing in are very vicious and nasty”* [(68), p.580].

Thus, the literature has not identified a best practice, regarding the single-sex and mixed-sex ward possibilities and the appropriateness of either should be determined on case-by-case basis. Additionally, women report that the physical layout and use of the *forensic* unit space is a key element contributing to feeling of safety or the lack thereof (68).

#### 4.5.4 Therapeutic activities

Across many studies, women hospitalized in psychiatric units expressed a desire for therapeutic activities and described their positive impacts when available (25, 29, 49, 59). Group-based interventions appeared to be both valued and beneficial for women, and Walsh-Harrington et al. (84) reported particularly positive perceptions of Dialectical Behaviour Therapy (DBT) among women psychiatric inpatients, regardless of their specific psychiatric diagnosis (59). DBT was also highlighted by Black American women hospitalized in psychiatric units in Creswell’s (27/30) article. In addition, other women emphasized both their wish for and the benefits of having opportunities to engage in self-care or other meaningful activities (52, 82).

#### 4.5.5 Integrating women’s voices

Integrating the lived experience of women service users can transform psychiatric care into a collaborative practice (86). Women with hospitalization histories can provide critical perspectives on iatrogenic trauma, gendered dynamics and unmet needs, informing staff training and tailored interventions (49). This peer-derived wisdom exposes how institutional routines perpetuate violence, paving the way for alternatives like dedicated spaces or trauma-sensitive approaches (54, 66).

#### 4.5.6 Trauma-informed and intersectional training

Training programs for psychiatric staff on trauma-informed care, intersectionality and gender-specific approaches to address women’s unique needs in inpatient settings are recommended by several authors (30, 49, 54). Trauma-sensitive practices can include physical interventions that respect survivors’ boundaries, such as carefully placing hands to avoid abuse-related trigger zones or therapies that restore humanity rather than objectification, helping women feel recognized as individuals rather than clinical cases.

A woman stated: *“They were very adamant on where they placed their hands because they knew the abuse… they’d know like which parts of my body not to touch”* [(30), p.382].

Proposed intersectional training examines can possibly address how gender interacts with race, gender and sexual orientation can shape care experiences with some themes like reproductive health, violence histories and relational dynamics (59, 85). These comprehensive programs could possibly reduce iatrogenic harm and equip staff to deliver equitable care that acknowledges women’s diverse trauma backgrounds and systemic vulnerabilities (48, 85).

## 5 Discussion

Our results highlight that women report various gender-based violences during hospitalisation and explore current or potential individual, interpersonal and systemic initiatives to prevent or address gender based-violence.

### 5.1 Intersectionality and differing psychiatric experiences

Many women in psychiatric setting live with multiple intersecting identities. Each of these identities and experiences interact and impact the care received. Despite growing recognition of intersectional experiences in research, studies fail to address that intersecting identities reflect interconnected forms of structural inequities in society (87). Empirical research tends to examine identities separately, without accounting for how the synergetic effects of each constitutes and culminates in the lived experience of the patient (87).

For women in psychiatry, these intersecting identities are not abstract constructs but more often lived realities that generate a sense of isolation and fear. Qualitative studies report that women in mental health settings report feeling powerless, unheard, misunderstood and alone (49). Thus, women do not experience these identities as analytically distinct, but rather a global experience –dehumanized and partaking in a “broken” system– of being positioned as the “other” within the system, shaped by what may feel like a “chain of events beyond their control” (30, 49).

### 5.2 From historical pathologization to EDI norms

Intersectionality often encompasses different identities, accounting for gender-based diversities, touching upon an individual level, whereas equity, diversity and inclusion (EDI) norms tend to aim for larger systemic oppressions on an organizational level (88). Psychiatric norms have historically reflected societal moral values, pathologizing women’s deviation from projected ideals (e.g., hysteria as uterine disorder). Historically, psychiatry took the place of both a moral and medical enterprise, reinforcing existing gender hierarchies (89). Women were pathologized if they deviated from societal expectations of “womanhood” (24). However, pathologization goes beyond only women’s perspectives. For example, pathologization also affected sexual orientation with homosexuality being identified as a mental illness in the Diagnostic and Statistical Manual of Mental Disorders 1 (90). Similar to women’s societal norms, this classification reflected a broader societal movement and values, such as heterosexism ideas. Thus, psychiatry evolves within different societal institutions.

Although psychiatry has reformed its methods and ethical premises, remnants of the historical view of women and mental illness still persist to this day and continue to shape the experiences of women. Women continue to report a differentiated psychiatric experience, with norms closely tied to patriarchal norms (47). But as psychiatric discourse often follows societal moral values, there has been increasing attention in prioritizing EDI in recent years (103). EDI norms in psychiatry focus on ethical and fair access to treatment, recognizing that diverse groups or communities do not inherently have the same access to support as others and present differing needs (91). Although EDI holds an essential role in policies and in the academic literature, the specific issue of women often seems less visible within these frameworks. Feminist movements over the past decades have profoundly impacted societal views on gender equality, which might suggest that nowadays women in psychiatric care receive the same access to care as men. However, our review of the recent literature identifies that women still operate in a system built for men. Indeed, EDI norms often present a systemic and institutional component, steering away from the everyday realities of women in psychiatry. Thus, Kelly and colleagues (88) posit that EDI, intersectionality and health equity are concepts that can be viewed as complimentary.

### 5.3 Coercion and women

Formal coercion, such as physical and chemical restraints, seclusion, and involuntary hospitalization, was widely discussed, and some women emphasized seclusion and restraints as mechanisms of control that reproduce broader societal dynamics; these were identified as particularly distressing experiences for women, especially when initiated by male staff (54, 59, 60). Consequently, women highlighted other forms of coercion, namely informal coercion, which Billé et al. (92) define as encompassing various techniques including persuasion, negotiation, interpersonal leverage, inducements, restrictions, blackmail, deception, threats, and witnessing displays of force. Women seem particularly sensitive to this more subtle form of coercion, including the overall coercive environment in psychiatric units during hospitalization (52, 53, 56, 66). This integrative review underscores the therapeutic relationship, interpersonal interactions, and care settings as potential factors influencing women’s experiences, feelings of safety, and sense of value.

### 5.4 Perinatal mental health

Postpartum mental health challenges, including hormonal fluctuations, adaptation to maternal roles, and associated psychosocial stressors, warrant consideration within general psychiatric units (49, 58). Although this review excluded specialized mother-baby psychiatric units, many hospitals lack such dedicated facilities and clinical reflection on these needs, resulting in these issues emerging across standard psychiatric wards (71). The actual reviewed literature demonstrates limited coverage of perinatal experiences, although their implications for gender-specific care could usefully inform the discussion section to highlight gaps in current practice and avenues for future research.

### 5.5 Menopause and perimenopause

Following the completion of this integrative review, there appears to be a significant lack of knowledge on the experiences of women hospitalized in psychiatry that specifically address perimenopause or menopause. This dimension can substantially influence their psychological lived experience, reported symptoms, relationships with healthcare providers, and the interpretation of their needs in the hospital setting (91, 93). The absence of this perspective in the literature perpetuates the invisibility of a highly relevant clinical reality associated with female gender, particularly given how hormonal fluctuations, somatic changes, and identity challenges during this period intersect with psychological distress (91, 93) Integrating this dimension would enhance comprehension of the overall experience of hospitalized women and the development of interventions more sensitive to gender and the life cycle (93).

### 5.6 Trauma-informed care: ideals and implementation gaps

The findings of this integrative review highlight that women seeking psychiatric care present specific needs and often complex life trajectories, embedded in trauma and difficult interpersonal relationships. Research shows that the healthcare system can inadvertently contribute to a re-traumatization in patients through coercion, such as seclusion, restraint, surveillance, restrictive rules, often exacerbating already open wounds. Traumatic events span individual, intergenerational, and historical experiences and do not only constitute patients’ past but frequently occur within the healthcare settings, in both general and forensic psychiatry (85, 94). Moreover, in settings prioritizing security, trauma responses can be interpreted as signs of dangerousness, rather than adaptive and coping responsive to an environment experienced as threatening and unstable. In secure settings for example, women can withhold information about their traumatic experiences in order to maintain autonomy and limit vulnerability (95). Being open about these experiences can lead to labels such as “dangerous” or “unstable” in these settings, further intensifying the distrust in the care team. These findings also highlight the need to consider women’s own perspectives on whether, and under what conditions, they want to be asked about trauma.

Over the past years, research has identified trauma-informed care as a valuable approach for women hospitalized in psychiatry (96). Trauma-informed care has become a theoretically endorsed model, promoting empowerment, security and trust between the patient and the caregiver (97, 98). While trauma-informed care is recognized as a best practice, empirical evidence supporting its implementation in mental healthcare remains limited. Dated conceptions of mental distress, limited resources, organizational barriers and priority on security over recovery often hinder trauma-informed care from being fully implemented in psychiatric settings (99, 100).

Furthermore, this issue is exacerbated by the lack of suitable trauma-informed care training for staff, with clinicians reporting feeling like they have insufficient knowledge and confidence to apply these principles in their practices (99). Clinicians explain feeling scared to ask questions about trauma to patients, due to the fear of potentially retraumatizing the women (30). For example, mental health nurses in Australia reportedly struggled with the implementation of trauma- informed care, beyond diminishing control practices (98). This underscores the importance of integrating women’s voices as a lever for change, for example by organizing women-only support activities on the unit, ensuring the presence of a female peer support worker, and co-designing trauma-informed practices with women who have lived experience.

Altogether, these factors highlight the existing gap between theoretical knowledge and clinical implementation about trauma- informed care, highlighting the need to continue developing guidelines and trainings that are easily accessible for clinicians in their everyday practice.

### 5.7 Multi-levels of change

Women’s differing experiences of care in psychiatry reflect broader systemic inequalities, rooted in historical ideals and societal norms. Diverse strategies operating on multiple levels hold potential to enhance psychiatric experiences for women. On an individual level, empathy can help foster gender-sensitive approaches, while recognizing women’s trauma histories prior to hospitalization and during hospitalization. On an environmental level, trauma-informed care and redesign patient spaces in order to foster safety, agency and trust between practitioners and patients. Finally, on an institutional level, EDI norms complemented by an intersectional approach could start a bigger conversation about accountability for patients beyond rhetorical commitments.

### 5.8 Strengths and limitations

A strength of this integrative review is that, to our knowledge, this is the first knowledge synthesis on this topic conducted by women with experiential knowledge (A. L., E. H., Women and Psychiatry Committee), which increases the rigor of the study by grounding it in lived experience. Furthermore, this integrative review tries to highlight systemic, collective and individual initiatives to counter gender-based violence that can be implemented in psychiatric settings.

Some intersectional identities of the women and settings that could have fostered an intersectional perspective were excluded, including women with substance use disorder, veterans, community settings, and outpatient services. Women’s experiences are often studied as whole without considering identity specificity; the lack of sociodemographic data of the study sample in most articles, as well as the limited number of articles explore specific identity sub-groups limits the use of a rigorous intersectional analysis framework. Therefore, just naming the hospital unit’s context – largely specific to the Western culture – limits the inclusion of some articles from other parts of the world where mental health issues are not taking care in a traditional hospital setting. The included languages (French and English) limited access to some women’s experiences. Given the breadth of topics covered in this article, some themes were addressed more briefly; this limitation, inherent to the format of a single paper, should be acknowledged, and future studies would benefit from exploring each theme in greater detail.

## 6 Conclusion

The experiences of hospitalized women receiving psychiatric care are at the crossroads of many intersecting and compounding identities and realities. These women often come from difficult backgrounds and psychiatric care can reinforce existing difficulties and vulnerabilities, if interventions are not tailored to women’s specific experiences. Thus, adequate training of staff and sustained research onto the lived realities of women receiving support both in general and forensic psychiatry is essential in ensuring women receive appropriate and beneficial care, corresponding to their treatment needs. Beyond its scientific contribution, carrying out this integrative review is grounded in a deep desire for change and empowerment. This integrative review was led by women who hold deeply personal knowledge of psychiatric hospitalization and who seek to transform these lived experiences into more just and responsive care for others.
