# Supplementary material for: The system wasn’t built for her: an integrative review of women’s experiences in psychiatric and forensic units
Source: Front Public Health. 2026 Jun 4;14:1810224. doi: 10.3389/fpubh.2026.1810224 (PMC13275366; doi:10.3389/fpubh.2026.1810224)
Supplement: Supplementary file 1 [file Supplementary_file_1.docx]

Supplementary Material 1. Full electronic search strategies (databases, search strings, filters, dates, and deduplication)

**Women’s experiences in psychiatric hostpizalition**

**Research strategy and numbers**

**PUBMED**

(("Women"[Mesh:NoExp] OR "Feminism"[Mesh] OR "Women’s rights"[Mesh] OR "Menstruation"[Mesh] OR "Pregnant People"[Mesh] OR "Pregnancy"[Mesh] OR "Postpartum Period"[Mesh] OR "Mothers"[Mesh:NoExp] OR "Reproductive Health"[Mesh] OR "Contraception"[Mesh] OR "Contraceptive Agents"[Mesh] OR "Contraceptive Devices"[Mesh] OR Women[TIAB] OR Woman[TIAB] OR Female*[TIAB] OR Feminis*[TIAB] OR Gender*[TIAB] OR Menstruation*[TIAB] OR Menstrual[TIAB] OR Menstruate[TIAB] OR Gynecological[TIAB] OR gynaecological[TIAB] OR Pregnant[TIAB] OR Pregnancy[TIAB] OR Postpartum[TIAB] OR Post-partum[TIAB] OR Mother*[TIAB] OR "reproductive health"[TIAB] OR contraception[TIAB] OR contraceptive*[TIAB]) **AND** ("Hospitals, Psychiatric"[Mesh] OR "Psychiatric Department, Hospital"[Mesh] OR (("Inpatients"[Mesh] OR "Hospitalization"[Mesh] OR "Persons with Psychiatric Disorders"[Mesh]) AND ("Mental Disorders"[Mesh:NoExp] OR "Psychiatry"[Mesh])) OR "Emergency Services, Psychiatric"[Mesh:NoExp] OR "Psychiatric hospital"[TIAB:~2] OR "psychiatric hospitals"[TIAB:~2] OR "Psychiatric hospitalisation"[TIAB:~2] OR "psychiatric hospitalisations"[TIAB:~2] OR "psychiatric hospitalization"[TIAB:~2] OR "psychiatric hospitalizations"[TIAB:~2] OR "Psychiatric unit"[TIAB:~2] OR "psychiatric units"[TIAB:~2] OR "Psychiatric ward"[TIAB:~2] OR "psychiatric wards"[TIAB:~2] OR "Psychiatric department"[TIAB:~2] OR "psychiatric departments"[TIAB:~2] OR "Psychiatric facility"[TIAB:~2] OR "psychiatric facilities"[TIAB:~2] OR "Psychiatric setting"[TIAB:~2] OR "psychiatric settings"[TIAB:~2] OR "Psychiatric institution"[TIAB:~2] OR "psychiatric institutions"[TIAB:~2] OR "Psychiatric inpatient"[TIAB:~2] OR "psychiatric inpatients"[TIAB:~2] OR "Psychiatric patient"[TIAB:~2] OR "psychiatric patients"[TIAB:~2] OR "mental health hospital"[TIAB:~2] OR "mental health hospitals"[TIAB:~2] OR "mental health hospitalisation"[TIAB:~2] OR "mental health hospitalisations"[TIAB:~2] OR "mental health hospitalization"[TIAB:~2] OR "mental health hospitalizations"[TIAB:~2] OR "mental health unit"[TIAB:~2] OR "mental health units"[TIAB:~2] OR "mental health ward"[TIAB:~2] OR "mental health wards"[TIAB:~2] OR "mental health department"[TIAB:~2] OR "mental health departments"[TIAB:~2] OR "mental health facility"[TIAB:~2] OR "mental health facilities"[TIAB:~2] OR "mental health setting"[TIAB:~2] OR "mental health settings"[TIAB:~2] OR "mental health institution"[TIAB:~2] OR "mental health institutions"[TIAB:~2] OR "mental health inpatient"[TIAB:~2] OR "mental health inpatients"[TIAB:~2] OR "mental health patient"[TIAB:~2] OR "mental health patients"[TIAB:~2] OR "psychiatric emergency"[TIAB:~2] OR "mental health emergency"[TIAB:~2])) **AND** ("Attitude"[Mesh:NoExp] OR "Attitude of Health Personnel"[Mesh:NoExp] OR "Refusal to Treat"[Mesh] OR "Stereotyping"[Mesh] OR "Bias"[Mesh:NoExp] OR "Social Stigma"[Mesh] OR "Sexism"[Mesh] OR "lived experience"[TIAB:~2] OR "lived experiences"[TIAB:~2] OR "patient experience"[TIAB:~3] OR "patient experiences"[TIAB:~3] OR "patients experience"[TIAB:~3] OR "patients experiences"[TIAB:~3] OR "user experience"[TIAB:~3] OR "user experiences"[TIAB:~3] OR "users experience"[TIAB:~3] OR "users experiences"[TIAB:~3] OR "women experience"[TIAB:~3] OR "women experiences"[TIAB:~3] OR "woman experience"[TIAB:~3] OR "woman experiences"[TIAB:~3] OR "female experience"[TIAB:~3] OR "female experiences"[TIAB:~3] OR "staff experience"[TIAB:~3] OR "staff experiences"[TIAB:~3] OR "nurse experience"[TIAB:~3] OR "nurse experiences"[TIAB:~3] OR "nurses experience"[TIAB:~3] OR "nurses experiences"[TIAB:~3] OR "patient experienced"[TIAB:~3] OR "patients experienced"[TIAB:~3] OR "user experienced"[TIAB:~3] OR "users experienced"[TIAB:~3] OR "women experienced"[TIAB:~3] OR "woman experienced"[TIAB:~3] OR "female experienced"[TIAB:~3] OR "staff experienced"[TIAB:~3] OR "nurse experienced"[TIAB:~3] OR "nurses experienced"[TIAB:~3] OR "patient perception"[TIAB:~3] OR "patient perceptions"[TIAB:~3] OR "patients perception"[TIAB:~3] OR "patients perceptions"[TIAB:~3] OR "user perception"[TIAB:~3] OR "user perceptions"[TIAB:~3] OR "users perception"[TIAB:~3] OR "users perceptions"[TIAB:~3] OR "women perception"[TIAB:~3] OR "women perceptions"[TIAB:~3] OR "woman perception"[TIAB:~3] OR "woman perceptions"[TIAB:~3] OR "female perception"[TIAB:~3] OR "female perceptions"[TIAB:~3] OR "staff perception"[TIAB:~3] OR "staff perceptions"[TIAB:~3] OR "nurse perception"[TIAB:~3] OR "nurse perceptions"[TIAB:~3] OR "nurses perception"[TIAB:~3] OR "nurses perceptions"[TIAB:~3] OR "patient perceived"[TIAB:~3] OR "patients perceived"[TIAB:~3] OR "user perceived"[TIAB:~3] OR "users perceived"[TIAB:~3] OR "women perceived"[TIAB:~3] OR "woman perceived"[TIAB:~3] OR "female perceived"[TIAB:~3] OR "staff perceived"[TIAB:~3] OR "nurse perceived"[TIAB:~3] OR "nurses perceived"[TIAB:~3] OR "patient view"[TIAB:~3] OR "patient views"[TIAB:~3] OR "patients view"[TIAB:~3] OR "patients views"[TIAB:~3] OR "user view"[TIAB:~3] OR "user views"[TIAB:~3] OR "users view"[TIAB:~3] OR "users views"[TIAB:~3] OR "women view"[TIAB:~3] OR "women views"[TIAB:~3] OR "woman view"[TIAB:~3] OR "woman views"[TIAB:~3] OR "female view"[TIAB:~3] OR "female views"[TIAB:~3] OR "staff view"[TIAB:~3] OR "staff views"[TIAB:~3] OR "nurse view"[TIAB:~3] OR "nurse views"[TIAB:~3] OR "nurses view"[TIAB:~3] OR "nurses views"[TIAB:~3] OR "patient perspective"[TIAB:~3] OR "patient perspectives"[TIAB:~3] OR "patients perspective"[TIAB:~3] OR "patients perspectives"[TIAB:~3] OR "user perspective"[TIAB:~3] OR "user perspectives"[TIAB:~3] OR "users perspective"[TIAB:~3] OR "users perspectives"[TIAB:~3] OR "women perspective"[TIAB:~3] OR "women perspectives"[TIAB:~3] OR "woman perspective"[TIAB:~3] OR "woman perspectives"[TIAB:~3] OR "female perspective"[TIAB:~3] OR "female perspectives"[TIAB:~3] OR "staff perspective"[TIAB:~3] OR "staff perspectives"[TIAB:~3] OR "nurse perspective"[TIAB:~3] OR "nurse perspectives"[TIAB:~3] OR "nurses perspective"[TIAB:~3] OR "nurses perspectives"[TIAB:~3] OR "attitude staff"[TIAB:~3] OR "attitudes staff"[TIAB:~3] OR "attitude nurse"[TIAB:~3] OR "attitudes nurse"[TIAB:~3] OR "attitude nurses"[TIAB:~3] OR "attitudes nurses"[TIAB:~3] OR stereotyp*[TIAB] OR stigma*[TIAB] OR "refusal to treat"[TIAB] OR bias[TIAB] OR feeling*[TIAB] OR sentiment*[TIAB] OR sexism[TIAB])

*Filtres : English, French*

**= 1908 résultats (30 avril 2025)**

**Medline**

1. Women/

2. feminism/ or women's rights/

3. Menstruation/

4. Pregnant People/

5. exp Pregnancy/

6. exp Postpartum Period/

7. Mothers/

8. Reproductive Health/

9. exp Contraception/

10. exp contraceptive agents/

11. exp Contraceptive Devices/

12. (Wom#n or Female* or Feminis* or Gender* or Menstruation* or Menstrual or Menstruate or Gyn?ecological or gynaecological or Pregnant or Pregnancy or Postpartum or Post-partum or Mother* or "reproductive health" or contraception or contraceptive*).ti,ab.

13. or/1-12

14. Hospitals, Psychiatric/

15. Psychiatric Department, Hospital/

16. Inpatients/

17. exp Hospitalization/

18. or/16-17

19. Mental Disorders/

20. exp Psychiatry/

21. Persons with Psychiatric Disorders/

22. or/19-21

23. and/18,22

24. Emergency Services, Psychiatric/

25. or/14-15,23-24

26. (((psychiatric or "mental health") adj2 (hospital* or unit* or ward* or department* or facility or facilities or setting* or institution* or inpatient* or patient*)) or ((psychiatric or "mental health") adj2 emergency)).ti,ab.

27. or/25-26

28. attitude/ or "attitude of health personnel"/ or stereotyping/

29. exp Refusal to Treat/

30. bias/

31. social stigma/ or sexism/

32. ((lived adj2 experience*) or ((patient* or user* or wom#n or female or staff or nurse*) adj3 (experience* or perception* or perceived or view or views or perspective*)) or (Attitude* adj3 (staff or nurse*)) or stereotyp* or stigma* or "refusal to treat" or bias or feeling* or sentiment* or sexism).ti,ab.

33. or/28-32

34. and/13,27,33

35. limit 34 to (english or french)

**= 1401 résultats (30 avril 2025)**

**EMBASE**

1. *female/ or exp *female by marital status/ or exp *female by occupation/ or exp *female by sexual orientation/

2. feminism/

3. women's rights/

4. menstruation/

5. pregnant woman/

6. exp pregnancy/

7. mother/

8. puerperium/

9. reproductive health/

10. exp contraception/

11. exp contraceptive agent/

12. contraceptive device/ or exp female contraceptive device/

13. (Wom#n or Female* or Feminis* or Gender* or Menstruation* or Menstrual or Menstruate or Gyn?ecological or gynaecological or Pregnant or Pregnancy or Postpartum or Post-partum or Mother* or "reproductive health" or contraception or contraceptive*).ti,ab.

14. or/1-13

15. mental hospital/

16. exp psychiatric department/

17. hospital patient/

18. hospitalization/

19. or/17-18

20. mental disease/

21. exp psychiatry/

22. mental patient/

23. or/20-22

24. and/19,23

25. psychiatric emergency service/

26. or/15-16,24-25

27. (((psychiatric or "mental health") adj2 (hospital* or unit* or ward* or department* or facility or facilities or setting* or institution* or inpatient* or patient*)) or ((psychiatric or "mental health") adj2 emergency)).ti,ab.

28. or/26-27

29. attitude/ or gender bias/ or health personnel attitude/

30. exp patient abandonment/

31. stereotyping/

32. cognitive bias/ or prejudice/ or exp sexism/ or exp gender based violence/

33. stigma/

34. social stigma/

35. personal experience/

36. ((lived adj2 experience*) or ((patient* or user* or wom#n or female or staff or nurse*) adj3 (experience* or perception* or perceived or view or views or perspective*)) or (Attitude* adj3 (staff or nurse*)) or stereotyp* or stigma* or "refusal to treat" or bias or feeling* or sentiment* or sexism).ti,ab.

37. or/29-36

38. and/14,28,37

39. limit 38 to ((english or french) and (article or article in press or books or chapter or "preprint (unpublished, non-peer reviewed)" or "review"))

**= 1656 results (30^th^ april 2025)**

**PsycINFO**

1. human females/ or exp mothers/ or female attitudes/ or women's rights/

2. feminism/

3. menstruation/ or reproductive health/

4. pregnancy/

5. birth control/ or exp contraceptive devices/ or exp reproductive rights/

6. oral contraceptives/

7. (Wom#n or Female* or Feminis* or Gender* or Menstruation* or Menstrual or Menstruate or Gyn?ecological or gynaecological or Pregnant or Pregnancy or Postpartum or Post-partum or Mother* or "reproductive health" or contraception or contraceptive*).ti,ab.

8. or/1-7

9. psychiatric hospitals/ or psychiatric units/

10. exp psychiatric hospitalization/ or exp psychiatric hospital admission/

11. hospitalized patients/

12. psychiatric patients/

13. hospitalization/

14. mental disorders/

15. exp psychiatry/

16. or/11-13

17. or/14-15

18. and/16-17

19. exp emergency services/

20. and/17,19

21. (((psychiatric or "mental health") adj2 (hospital* or unit* or ward* or department* or facility or facilities or setting* or institution* or inpatient* or patient*)) or ((psychiatric or "mental health") adj2 emergency)).ti,ab.

22. or/9-10,18,20-21

23. lived experience/

24. attitudes/ or caregiver attitudes/ or female attitudes/ or gender role attitudes/ or exp sex role attitudes/ or stereotyped attitudes/ or exp prejudice/ or exp stigma/

25. gender bias/ or exp gender violence/ or sexism/ or sex discrimination/

26. ((lived adj2 experience*) or ((patient* or user* or wom#n or female or staff or nurse*) adj3 (experience* or perception* or perceived or view or views or perspective*)) or (Attitude* adj3 (staff or nurse*)) or stereotyp* or stigma* or "refusal to treat" or bias or feeling* or sentiment* or sexism).ti,ab.

27. or/23-26

28. and/8,22,27

29. limit 28 to (("0100 journal" or "0200 book" or "0400 dissertation abstract") and (english or french))

**= 1586 results (30^th^ april 2025)**

**EBM Reviews – Cochrane Database of Systematic Reviews**

1. (Wom#n or Female* or Feminis* or Gender* or Menstruation* or Menstrual or Menstruate or Gyn?ecological or gynaecological or Pregnant or Pregnancy or Postpartum or Post-partum or Mother* or "reproductive health" or contraception or contraceptive*).ti,ab.

2. (((psychiatric or "mental health") adj2 (hospital* or unit* or ward* or department* or facility or facilities or setting* or institution* or inpatient* or patient*)) or ((psychiatric or "mental health") adj2 emergency)).ti,ab.

3. ((lived adj2 experience*) or ((patient* or user* or wom#n or female or staff or nurse*) adj3 (experience* or perception* or perceived or view or views or perspective*)) or (Attitude* adj3 (staff or nurse*)) or stereotyp* or stigma* or "refusal to treat" or bias or feeling* or sentiment* or sexism).ti,ab.

4. and/1-3

**= 3 results (30^th^ april 2025)**

**CINAHL**

|  |  |
| --- | --- |
| S13 | S3 AND S9 AND S12  Limiters - Language: English, French; Publication Type: Book, Book Chapter, Doctoral Dissertation, Journal Article, Review |
| S12 | S10 OR S11 |
| S11 | TI ( (lived N2 experience*) OR ((patient* OR user* OR wom#n OR female OR staff OR nurse*) N3 (experience* OR perception* OR perceived OR view OR views OR perspective*)) OR (Attitude* N3 (staff OR nurse*)) OR stereotyp* OR stigma* OR "refusal to treat" OR bias OR feeling* OR sentiment* OR sexism ) OR AB ( (lived N2 experience*) OR ((patient* OR user* OR wom#n OR female OR staff OR nurse*) N3 (experience* OR perception* OR perceived OR view OR views OR perspective*)) OR (Attitude* N3 (staff OR nurse*)) OR stereotyp* OR stigma* OR "refusal to treat" OR bias OR feeling* OR sentiment* OR sexism ) |
| S10 | (MH "Life Experiences") OR (MH "Attitude") OR (MH "Attitude of Health Personnel+") OR (MH "Gender Bias") OR (MH "Patient Attitudes") OR (MH "Caregiver Attitudes") OR (MH "Refusal to Treat") OR (MH "Patient Abandonment") OR (MH "Stereotyping") OR (MH "Stigma") OR (MH "Prejudice") OR (MH "Sexism") OR (MH "Implicit Bias") OR (MH "Gender-Based Violence") |
| S9 | S4 OR S7 OR S8 |
| S8 | TI ( ((psychiatric OR "mental health") N2 (hospital* OR unit* OR ward* OR department* OR facility OR facilities OR setting* OR institution* OR inpatient* OR patient*)) OR ((psychiatric OR "mental health") N2 emergency) ) OR AB ( ((psychiatric OR "mental health") N2 (hospital* OR unit* OR ward* OR department* OR facility OR facilities OR setting* OR institution* OR inpatient* OR patient*)) OR ((psychiatric OR "mental health") N2 emergency) ) |
| S7 | S5 AND S6 |
| S6 | (MH "Mental Disorders") OR (MH "Psychiatry+") OR (MH "Persons with Mental Disorders") |
| S5 | (MH "Inpatients") OR (MH "Hospitalization") |
| S4 | (MH "Hospitals, Psychiatric") OR (MH "Psychiatric Units") OR (MH "Psychiatric Emergencies") |
| S3 | S1 OR S2 |
| S2 | TI ( Wom#n OR Female* OR Feminis* OR Gender* OR Menstruation* OR Menstrual OR Menstruate OR Gyn?ecological OR gynaecological OR Pregnant OR Pregnancy OR Postpartum OR Post-partum OR Mother* OR "reproductive health" OR contraception OR contraceptive* ) OR AB ( Wom#n OR Female* OR Feminis* OR Gender* OR Menstruation* OR Menstrual OR Menstruate OR Gyn?ecological OR gynaecological OR Pregnant OR Pregnancy OR Postpartum OR Post-partum OR Mother* OR "reproductive health" OR contraception OR contraceptive* ) |
| S1 | (MH "Women+") OR (MH "Feminism+") OR (MH "Women's Rights") OR (MH "Menstruation") OR (MH "Pregnancy") OR (MH "Puerperium") OR (MH "Mothers+") OR (MH "Reproductive Health") OR (MH "Contraception+") OR (MH "Contraceptive Devices+") OR (MH "Contraceptive Agents+") |

**= 1009 results (30th april 2025)**

**Number of identified results : 7563**

**Number of duplicated (identified through SR Accelerator) :** [**https://sr-accelerator.com/#/deduplicator**](https://sr-accelerator.com/#/deduplicator)**) : 3816**

**ADDED TOTAL : 3747**
