# Supplementary material for: The system wasn’t built for her: an integrative review of women’s experiences in psychiatric and forensic units
Source: Front Public Health. 2026 Jun 4;14:1810224. doi: 10.3389/fpubh.2026.1810224 (PMC13275366; doi:10.3389/fpubh.2026.1810224)
Supplement: Supplementary file 2 [file Supplementary_file_2.docx]

Supplementary material 2. Detailed conceptual framework for the systematic review on women’s expereinces during psychiatric hospitalziation

**Women’s experiences in psychiatric hospitalization**

**Concept plan**

| **Women** | **Psychiatric Hospitalization** | **Experiences** |
| --- | --- | --- |
| **Descriptors (MeSH)**  "Women"[Mesh:NoExp]  "Feminism"[Mesh]  "Women’s rights"[Mesh]  "Menstruation"[Mesh]  "Pregnant People"[Mesh]  "Pregnancy"[Mesh]  "Postpartum Period"[Mesh]  "Mothers"[Mesh:NoExp]  "Reproductive Health"[Mesh]  "Contraception"[Mesh]  "Contraceptive Agents"[Mesh]  "Contraceptive Devices"[Mesh]  **Keywords (title, abstract)**  Women  Woman  Female(s)  Feminist(s)  Feminism  Gender  Gendered  Menstruation(s)  Menstrual  Menstruate  Gyn(a)ecological  Pregnant  Pregnancy  Postpartum  Post-partum  Mother(s)  Motherhood  Reproductive health  Contraception  Contraceptive(s) | **Descriptors (MeSH)**  "Hospitals, Psychiatric"[Mesh]  "Psychiatric Department, Hospital"[Mesh]  "Inpatients"[Mesh]  "Hospitalization"[Mesh]  AND  "Mental Disorders"[Mesh:NoExp]  "Psychiatry"[Mesh]  "Persons with Psychiatric Disorders"[Mesh]  "Emergency Services, Psychiatric"[Mesh:NoExp]  **Keywords (title, abstract)**  Psychiatric hospital(s)  Psychiatric hospitalis(z)ation(s)  Psychiatric unit(s)  Psychiatric ward(s)  Psychiatric department(s)  Psychiatric facility(ies)  Psychiatric setting(s)  Psychiatric institution(s)  Psychiatric inpatient(s)  Inpatient psychiatric  Psychiatric patient(s)  Mental health hospital(s)  Mental health hospitalis(z)ation(s)  Mental health unit(s)  Mental health ward(s)  Mental health department(s)  Mental health facility(ies)  Mental health setting(s)  Mental health institution(s)  Mental health inpatient(s)  Inpatient mental health  Mental health patient(s)  Psychiatric emergency  Mental health emergency | **Descriptors (MeSH)**  "Attitude"[Mesh:NoExp]  "Attitude of Health Personnel"[Mesh:NoExp]  "Refusal to Treat"[Mesh]  "Stereotyping"[Mesh]  "Bias"[Mesh:NoExp]  "Social Stigma"[Mesh]  "Sexism"[Mesh]  **Keywords (title, abstract)**  Experience(s)  Experienced  Perception(s)  Perceived  View(s)  Perspective(s)  ADJ  Lived  Patient(s)  User(s)  Women  Woman  Female  Staff  Nurse(s)  Attitude(s)  ADJ  Staff  Nurse(s)  Stereotyping  Stereotype*  Stigma(s)  Stigmatization  Refusal to treat  Bias  Feelings  Sentiment(s)  Sexism |

English, french

Articles, thesis, books, book chapters

**Articles to aim for** :

<https://pubmed.ncbi.nlm.nih.gov/39435962/>

<https://pubmed.ncbi.nlm.nih.gov/19291494/>

<https://pubmed.ncbi.nlm.nih.gov/23686217/>

<https://pubmed.ncbi.nlm.nih.gov/33857788/>

39435962 OR 19291494 OR 23686217 OR 33857788

**Concept 1**

"Women"[Mesh:NoExp] OR "Feminism"[Mesh] OR "Women’s rights"[Mesh] OR "Menstruation"[Mesh] OR "Pregnant People"[Mesh] OR "Pregnancy"[Mesh] OR "Postpartum Period"[Mesh] OR "Mothers"[Mesh:NoExp] OR "Reproductive Health"[Mesh] OR "Contraception"[Mesh] OR "Contraceptive Agents"[Mesh] OR "Contraceptive Devices"[Mesh] OR Women[TIAB] OR Woman[TIAB] OR Female*[TIAB] OR Feminis*[TIAB] OR Gender*[TIAB] OR Menstruation*[TIAB] OR Menstrual[TIAB] OR Menstruate[TIAB] OR Gynecological[TIAB] OR gynaecological[TIAB] OR Pregnant[TIAB] OR Pregnancy[TIAB] OR Postpartum[TIAB] OR Post-partum[TIAB] OR Mother*[TIAB] OR "reproductive health"[TIAB] OR contraception[TIAB] OR contraceptive*[TIAB]

**Concept 2**

"Hospitals, Psychiatric"[Mesh] OR "Psychiatric Department, Hospital"[Mesh] OR (("Inpatients"[Mesh] OR "Hospitalization"[Mesh] OR "Persons with Psychiatric Disorders"[Mesh]) AND ("Mental Disorders"[Mesh:NoExp] OR "Psychiatry"[Mesh])) OR "Emergency Services, Psychiatric"[Mesh:NoExp] OR "Psychiatric hospital"[TIAB:~2] OR "psychiatric hospitals"[TIAB:~2] OR "Psychiatric hospitalisation"[TIAB:~2] OR "psychiatric hospitalisations"[TIAB:~2] OR "psychiatric hospitalization"[TIAB:~2] OR "psychiatric hospitalizations"[TIAB:~2] OR "Psychiatric unit"[TIAB:~2] OR "psychiatric units"[TIAB:~2] OR "Psychiatric ward"[TIAB:~2] OR "psychiatric wards"[TIAB:~2] OR "Psychiatric department"[TIAB:~2] OR "psychiatric departments"[TIAB:~2] OR "Psychiatric facility"[TIAB:~2] OR "psychiatric facilities"[TIAB:~2] OR "Psychiatric setting"[TIAB:~2] OR "psychiatric settings"[TIAB:~2] OR "Psychiatric institution"[TIAB:~2] OR "psychiatric institutions"[TIAB:~2] OR "Psychiatric inpatient"[TIAB:~2] OR "psychiatric inpatients"[TIAB:~2] OR "Psychiatric patient"[TIAB:~2] OR "psychiatric patients"[TIAB:~2] OR "mental health hospital"[TIAB:~2] OR "mental health hospitals"[TIAB:~2] OR "mental health hospitalisation"[TIAB:~2] OR "mental health hospitalisations"[TIAB:~2] OR "mental health hospitalization"[TIAB:~2] OR "mental health hospitalizations"[TIAB:~2] OR "mental health unit"[TIAB:~2] OR "mental health units"[TIAB:~2] OR "mental health ward"[TIAB:~2] OR "mental health wards"[TIAB:~2] OR "mental health department"[TIAB:~2] OR "mental health departments"[TIAB:~2] OR "mental health facility"[TIAB:~2] OR "mental health facilities"[TIAB:~2] OR "mental health setting"[TIAB:~2] OR "mental health settings"[TIAB:~2] OR "mental health institution"[TIAB:~2] OR "mental health institutions"[TIAB:~2] OR "mental health inpatient"[TIAB:~2] OR "mental health inpatients"[TIAB:~2] OR "mental health patient"[TIAB:~2] OR "mental health patients"[TIAB:~2] OR "psychiatric emergency"[TIAB:~2] OR "mental health emergency"[TIAB:~2]

**Concept 3**

"Attitude"[Mesh:NoExp] OR "Attitude of Health Personnel"[Mesh:NoExp] OR "Refusal to Treat"[Mesh] OR "Stereotyping"[Mesh] OR "Bias"[Mesh:NoExp] OR "Social Stigma"[Mesh] OR "Sexism"[Mesh] OR "lived experience"[TIAB:~2] OR "lived experiences"[TIAB:~2] OR "patient experience"[TIAB:~3] OR "patient experiences"[TIAB:~3] OR "patients experience"[TIAB:~3] OR "patients experiences"[TIAB:~3] OR "user experience"[TIAB:~3] OR "user experiences"[TIAB:~3] OR "users experience"[TIAB:~3] OR "users experiences"[TIAB:~3] OR "women experience"[TIAB:~3] OR "women experiences"[TIAB:~3] OR "woman experience"[TIAB:~3] OR "woman experiences"[TIAB:~3] OR "female experience"[TIAB:~3] OR "female experiences"[TIAB:~3] OR "staff experience"[TIAB:~3] OR "staff experiences"[TIAB:~3] OR "nurse experience"[TIAB:~3] OR "nurse experiences"[TIAB:~3] OR "nurses experience"[TIAB:~3] OR "nurses experiences"[TIAB:~3] OR "patient experienced"[TIAB:~3] OR "patients experienced"[TIAB:~3] OR "user experienced"[TIAB:~3] OR "users experienced"[TIAB:~3] OR "women experienced"[TIAB:~3] OR "woman experienced"[TIAB:~3] OR "female experienced"[TIAB:~3] OR "staff experienced"[TIAB:~3] OR "nurse experienced"[TIAB:~3] OR "nurses experienced"[TIAB:~3] OR "patient perception"[TIAB:~3] OR "patient perceptions"[TIAB:~3] OR "patients perception"[TIAB:~3] OR "patients perceptions"[TIAB:~3] OR "user perception"[TIAB:~3] OR "user perceptions"[TIAB:~3] OR "users perception"[TIAB:~3] OR "users perceptions"[TIAB:~3] OR "women perception"[TIAB:~3] OR "women perceptions"[TIAB:~3] OR "woman perception"[TIAB:~3] OR "woman perceptions"[TIAB:~3] OR "female perception"[TIAB:~3] OR "female perceptions"[TIAB:~3] OR "staff perception"[TIAB:~3] OR "staff perceptions"[TIAB:~3] OR "nurse perception"[TIAB:~3] OR "nurse perceptions"[TIAB:~3] OR "nurses perception"[TIAB:~3] OR "nurses perceptions"[TIAB:~3] OR "patient perceived"[TIAB:~3] OR "patients perceived"[TIAB:~3] OR "user perceived"[TIAB:~3] OR "users perceived"[TIAB:~3] OR "women perceived"[TIAB:~3] OR "woman perceived"[TIAB:~3] OR "female perceived"[TIAB:~3] OR "staff perceived"[TIAB:~3] OR "nurse perceived"[TIAB:~3] OR "nurses perceived"[TIAB:~3] OR "patient view"[TIAB:~3] OR "patient views"[TIAB:~3] OR "patients view"[TIAB:~3] OR "patients views"[TIAB:~3] OR "user view"[TIAB:~3] OR "user views"[TIAB:~3] OR "users view"[TIAB:~3] OR "users views"[TIAB:~3] OR "women view"[TIAB:~3] OR "women views"[TIAB:~3] OR "woman view"[TIAB:~3] OR "woman views"[TIAB:~3] OR "female view"[TIAB:~3] OR "female views"[TIAB:~3] OR "staff view"[TIAB:~3] OR "staff views"[TIAB:~3] OR "nurse view"[TIAB:~3] OR "nurse views"[TIAB:~3] OR "nurses view"[TIAB:~3] OR "nurses views"[TIAB:~3] OR "patient perspective"[TIAB:~3] OR "patient perspectives"[TIAB:~3] OR "patients perspective"[TIAB:~3] OR "patients perspectives"[TIAB:~3] OR "user perspective"[TIAB:~3] OR "user perspectives"[TIAB:~3] OR "users perspective"[TIAB:~3] OR "users perspectives"[TIAB:~3] OR "women perspective"[TIAB:~3] OR "women perspectives"[TIAB:~3] OR "woman perspective"[TIAB:~3] OR "woman perspectives"[TIAB:~3] OR "female perspective"[TIAB:~3] OR "female perspectives"[TIAB:~3] OR "staff perspective"[TIAB:~3] OR "staff perspectives"[TIAB:~3] OR "nurse perspective"[TIAB:~3] OR "nurse perspectives"[TIAB:~3] OR "nurses perspective"[TIAB:~3] OR "nurses perspectives"[TIAB:~3] OR "attitude staff"[TIAB:~3] OR "attitudes staff"[TIAB:~3] OR "attitude nurse"[TIAB:~3] OR "attitudes nurse"[TIAB:~3] OR "attitude nurses"[TIAB:~3] OR "attitudes nurses"[TIAB:~3] OR stereotyp*[TIAB] OR stigma*[TIAB] OR "refusal to treat"[TIAB] OR bias[TIAB] OR feeling*[TIAB] OR sentiment*[TIAB] OR sexism[TIAB]

**Vocabulaire libre – Autres bases de données**

Wom#n OR Female* OR Feminis* OR Gender* OR Menstruation* OR Menstrual OR Menstruate OR Gyn?ecological OR gynaecological OR Pregnant OR Pregnancy OR Postpartum OR Post-partum OR Mother* OR "reproductive health" OR contraception OR contraceptive*

((psychiatric OR "mental health") ADJ2 (hospital* OR unit* OR ward* OR department* OR facility OR facilities OR setting* OR institution* OR inpatient* OR patient*)) OR ((psychiatric OR "mental health") ADJ2 emergency)

(lived ADJ2 experience*) OR ((patient* OR user* OR wom#n OR female OR staff OR nurse*) ADJ3 (experience* OR perception* OR perceived OR view OR views OR perspective*)) OR (Attitude* ADJ3 (staff OR nurse*)) OR stereotyp* OR stigma* OR "refusal to treat" OR bias OR feeling* OR sentiment* OR sexism
